# Modification of comet-FISH technique by using temperature instead of chemical denaturation

**DOI:** 10.1016/j.mex.2014.08.010

**Published:** 2014-08-28

**Authors:** Marin Mladinic, Davor Zeljezic

**Affiliations:** Institute for Medical Research and Occupational Health, Ksaverska cesta 2, 10000 Zagreb, Croatia

**Keywords:** Comet-FISH, TP53, Temperature denaturation, Lymphocytes, Primary DNA damage, Gene fragmentation

## Abstract

Comet-FISH technique is an extension of commonly used comet assay. Its purpose is to determine whether primary DNA damage which comet assay detects occurred within a sequence of interest that is visualized by hybridization of fluorescent probe. Presence of the signal in comet tail indicates impaired structural integrity of sequence. Our modifications to the original comet-FISH technique described by Rapp et al. (2000) [1] include:

• increase in probe binding specificity,
• increased rate of successful hybridization,
• simultaneous temperature denaturation of both, slide and probe.

## Graphical abstract

**Figure:**
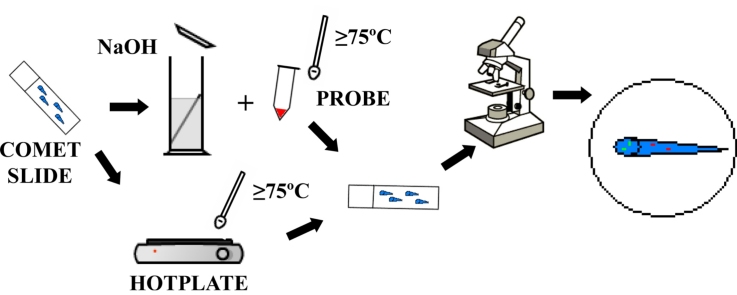

## Method details

Comet-FISH technique is an extension of commonly used comet assay. Its purpose is to determine whether primary damage which comet assay detects, affected specific gene (or other sequence) of interest [1]. Signal detected in the tail of the comet indicates damage to the DNA sequence of interest.

In short, comet-FISH technique consists of the following steps: (a) preparation of the comet assay slides; (b) performing the lysis, denaturation and electrophoresis; (c) performing fluorescent in situ hybridization on comet assay slides; (d) analysis of slides (evaluation of primary DNA damage and/or specific gene damage). In preparation of comet assay slides we used leukocytes from whole blood and isolated lymphocytes.

## Materials

1. Vertical or horizontal glass Coplin jars
2. Coverslips (18 mm × 18 mm, 22 mm × 22 mm and 24 mm × 40 mm)
3. Microscopic slides
4. Parafilm
5. Hot plate
6. Incubator
7. Water bath
8. Moist chamber (glass or plastic box with platform for slides above a layer of water)
9. Horizontal electrophoresis tank with power supply
10. Microwave oven
11. Epifluorescence microscope
12. Pasteur pipette

## Reagents and buffers

1. Normal melting point agarose (NMP agarose): 1% in redistilled H_2_O
2. Low melting point agarose (LMP agarose): 0.5% in PBS
3. Histopaque^®^ 1077 (or any other density gradient media for lymphocyte isolation)
4. Phosphate buffered saline (PBS) pH 7.4
5. RPMI-1640 medium
6. Lysis solution (stock): prepare 1 L of 2.5 M NaCl, 0.1 M Na_2_EDTA, 10 mM Trizma^®^ base, 1% N-lauroylsarcosine sodium salt. Set pH at 10 with 10 M NaOH solution. When preparing 100 ml of working lysis buffer use: 89 ml of stock lysis solution, 1 ml of Triton X-100 (1%) and 10 ml of 10% dimethyl sulfoxide (DMSO).
7. Alkaline denaturation and electrophoresis buffer: 0.1 M NaOH, 1 mM Na_2_EDTA.
8. Saline–sodium citrate buffer (20× SSC): 0.3 M sodium citrate, 3 M sodium chloride, pH 7.0
9. Tween-20
10. Photo glue
11. Aluminum foil

### Preparation of the comet assay slides

Regular microscope slides used for precoating should be grease-free, if necessary they can be cleaned by soaking in alcohol and wiping them dry with a clean tissue. Immerse slides in a Coplin jar with melted 1% NMP agarose. Wipe the back of the slide clean with a tissue, drain the surplus of agarose from the slide and air-dry it overnight at room temperature. Heat the 0.5% LMP agarose in a microwave oven, put it in a water bath at 37 °C and allow sufficient time to equilibrate to this temperature. For lymphocyte isolation, use 1:1 (v/v) Histopaque^®^ 1077 and whole blood. Centrifuge for 30 min at 400 × *g*. By using a Pasteur pipette collect the cells that remained at the interface (in between the clear histopaque and pink-yellow plasma layer) forming a “buffy coat” band. Resuspend collected cells in with PBS or RPMI-1640 medium and adjust the volume to 5 ml. Centrifuge again for 5 min at 250 × *g*. Remove supernatant, add PBS or RPMI to 1 ml and resuspend the cells. Place an aliquot of 1–10 μl (depending on the density of cells in suspension) in a microcentrifuge tube, add 100 μl of warmed 0.5% LMP agarose and mix by aspirating agarose up and down with pipette. Place 100 μl of cell suspension in agarose (use same pipette tip) on the precoated microscopic slide. Cover with a 24 × 40 mm coverslip. Leave slides on ice for 5–10 min until the agarose has polymerized.

Blood sampling was in accordance with the high ethical standards, complied with the principles laid down in the Declaration of Helsinki and approved by the Institutional Ethical committee.

### Lysis, denaturation and electrophoresis

Remove the coverslip and put the slides in a Coplin jar with cold (4 °C) freshly prepared working lysis solution for at least 1 h at 4 °C. Transfer the slides to another Coplin jar filed with chilled (4 °C) denaturation/electrophoresis buffer. Leave for 20 min. Place the slides in a horizontal electrophoresis tank containing chilled (4 °C) denaturation/electrophoresis buffer. Make sure that gels are covered with buffer. Alkaline treatment and electrophoresis are carried out at 4 °C. Electrophoresis should be run for 20 min at 300 mA and 0.7–1.0 V/cm, depending on the type of cells used. If there is too much electrolyte covering the slides, the designated voltage will not be reached. Stop the electrophoresis, remove some of the solution and run it again. Neutralize the slides by washing them in PBS in a Coplin jar for 10 min followed by additional 10 min wash in redistilled water. Afterwards, dehydrate the slides in a Coplin jar by washing in a series of ethanol (70%, 96%, 100%). Air-dry the slides.

### Fluorescent in situ hybridization of comet assay slides (comet-FISH)

In current protocols [2–5] slides were immersed for 30 min in absolute ethanol at 4 °C followed by 0.5 M NaOH for 25 min to ensure complete denaturation of DNA. Slides were dehydrated in rising series of alcohol, 5 min each, while probe was being denaturated separately in a water bath heated above 75 °C. We decided to modify and simplify the protocol. An innovative aspect of our modification is that instead of separated chemical denaturation of slides and temperature denaturation of probe, we applied simultaneous temperature denaturation for both slide and probe on a hot plate. Table 1 shows comparison of different variables between temperature and chemical denaturation in comet-FISH technique indicating improvements of our approach. In our experiments by using temperature denaturation we managed to increase the hybridization rate by 16% compared to chemical denaturation.

#### Slide and probe preparation

Following the comet assay, comet-FISH protocol begins by immersing air-dried comet slides in 2× SSC step in a Coplin jar for 5 min at room temperature. In this step, SSC is used to optimize the environment of the gels the nucleoids are in, which is needed to maximize the rate of probe annealing to the nucleic acid target and minimizes non-specific binding and cross-hybridization. Afterwards, dehydrate the slides again in a Coplin jar by washing in series of ethanols (70%, 96%, 100%), for 5 min each. The next step is denaturation, which is an essential for this technique to work. Both, slide and probe were separately prewarmed at 37 °C for 5 min. Place the 10 μl of warmed probe on the selected area of the slide, carefully apply a 18 mm × 18 mm coverslip.

#### Denaturation

Similar approach as used in classical FISH and paraffin embedded tissue sections was applied, considering that temperature co-denaturation does not affect sub-cellular structures. Co-denature the slide and probe on a hot plate at 75 °C for 2 min (according to manufacturer's instructions). Our slides were hybridized with “p53 deletion probe” (Cytocell, Cambridge, UK), containing Texas Red-labeled probe for TP53 gene and FITC-labeled probe for centromeric region of chromosome 17 (cen 17). However, we found out that better signals are obtained at higher temperatures than 75 °C and longer denaturation times. For instance, temperature could be raised up to 82 °C and up to 5 min of denaturation time depending on the manufacturer.

General presumption was that low melting point agarose normally used for making comet gels would melt at the temperature used for DNA denaturation in standard cytogenetic preparations (70 °C). However, in our experiments this is not the case. One of the reasons for this may be that prior to temperature denaturation our slides were completely dehydrated, and gels air-dried. Before conducting comet-FISH experiments we tried to melt dried comet slides at different temperatures. The highest temperature empirically tested at which agarose gel did not melt was 92 °C. In another publication temperature denaturation was performed at 80 °C for 2 min [6].

#### Hybridization

Seal the coverslip with photo glue and place the slides in a humid lightproof box (use aluminum foil to protect from light) for 1 h to overnight at 37 °C for hybridization. The hybridization of the probe to genome was better with an overnight incubation.

#### Post-hybridization washes

**Fig. 1:**
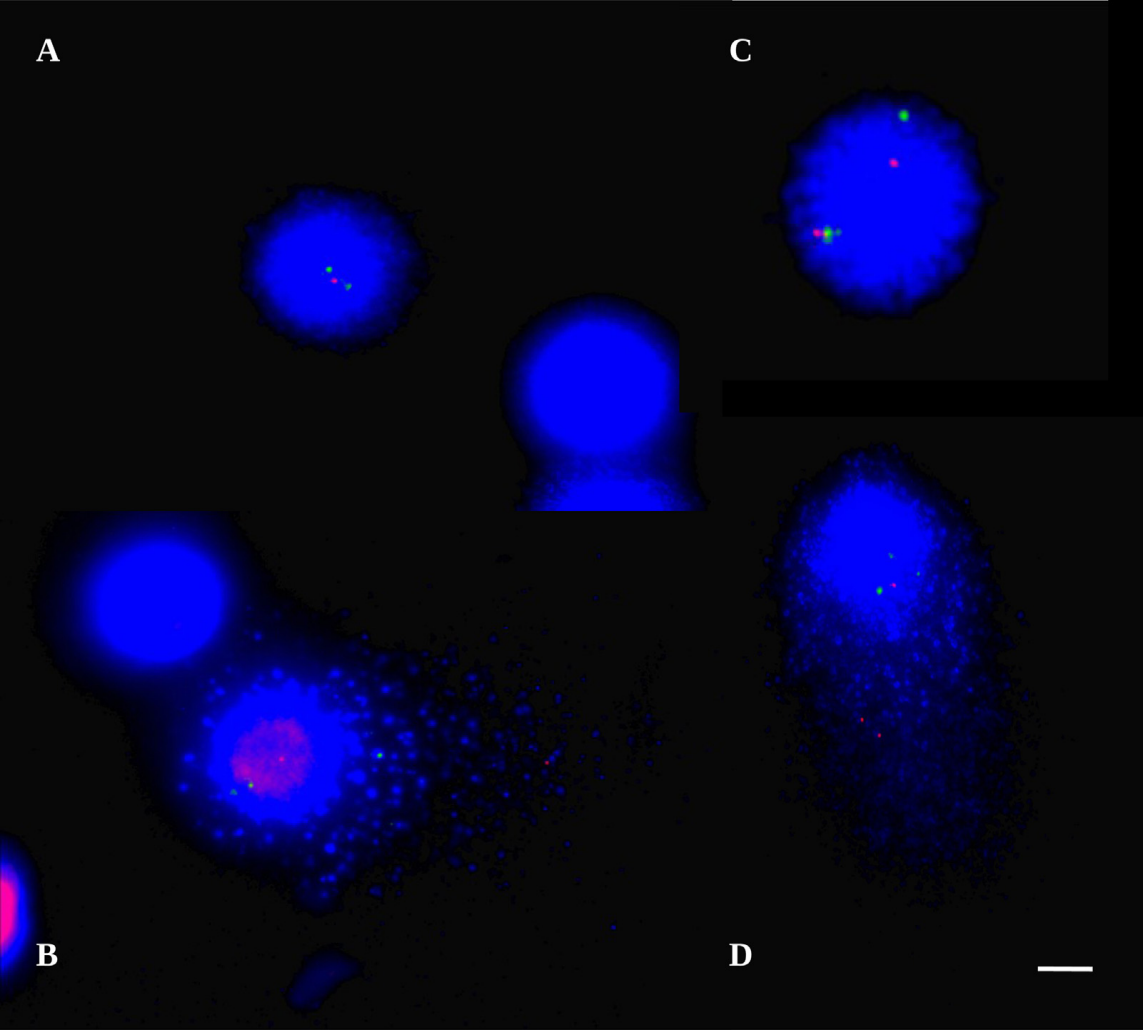
Comet-FISH images of lymphocytes hybridized with p53 probe (TP53-red signal, cen 17 green signal); Chemical denaturation is shown in (A) and (B) while temperature denaturation is shown in (C) and (D). Picture (A) represents undamaged nucleoids but hybridization rate is lower (no signals in second nucleoid). Picture (B) represent fragmented nucleoid with some non-specific hybridization and lower hybridization rate (again no signals in second nucleoid). Picture (C) represents undamaged nucleoid. Picture (D) represents fragmented nucleoid with successful hybridization; bar 10 μm.

Remove the glue carefully, and then the coverslip. Post-hybridization washes were done following the supplier's instructions which were 0.25× SC at 72 °C pH 7.0 for 2 min followed by 2× SSC, 0.05% Tween-20 at room temperature for 30 s. In this step SSC provides high ionic strength needed for non-specifically and cross-hybridized probe to denature and to be washed out from the slide. However, we found out that these post-hybridization washes were too stringent for comet-FISH procedure causing even probe hybridized to target sequences to dissociate and there were hardly any signals on the slides. Therefore, we decreased the stringency of the washes by increasing the concentration of the salt from 0.25× SSC to 2× SSC and decreasing the temperature to 65 °C for 1.45 min. It was followed by 2× SSC, 0.05% Tween-20 at room temperature for 30 s. Further, we dehydrated the slides by washing them in ice cold alcohols for 5 min each (70%, 96%, 100%). Slides were counterstained with DAPI prepared in antifade solution (Q Biogen, UK), covered with 22 × 22 mm coverslip, sealed with photo glue and analyzed by using an Olympus AX70 (Tokyo, Japan) epifluorescence microscope. Simultaneous co-denaturation of both slide and probe by temperature allowed better hybridization and visualization of signals in comet-FISH. Visual comparison between chemical and temperature denaturation can also be seen in images of nucleoids hybridized with TP53 probe using comet-FISH (Fig. 1).

## Reasoning behind using temperature denaturation

Experiments with classical FISH and FISH on histological cross-sections embedded in paraffin have shown that post hybridization washes with aqueous buffers of high ionic strength ranging from 45 to 70 °C did not lead to loss of cell structure. On the contrary, use of low ionic strength buffers would cause detachment of paraffin embedded tissues from the slide. We observed that in the comet-FISH assay chemical denaturation with NaOH, which has low ionic strength, affects the loss of gels from slides, presumably by disturbing the ionic/van der Waals bonds.

With regard to probe annealing, it has to be stressed that less concentrated salt solution, longer duration of washing or higher temperature will yield a higher stringency wash resulting in higher probe binding specificity to the DNA. The idea behind temperature denaturation also comes from earlier observations of FISH experiments on micronucleus preparations, where temperature denaturation is commonly used together with post hybridization washes at high temperatures. In our approach used here, we established that the temperature denaturation minimized the gel loss risk. Further, dehydration and heating of agarose gels did not result in loss of nucleoid structure on gels.

## Figures and Tables

**Table 1:** Comparative table of different variables between temperature and chemical denaturation in comet-FISH technique.

Variables	Temperature denaturation	Chemical denaturation
Toxicity^a^	Lower	Higher
Probe binding specificity	Higher	Lower
Hybridization success rate^b^	Higher (82.0 ± 6.0%)	Lower (66.0 ± 7.9%)
Signals vs. background contrast	Higher	Lower
Control of temperature transfer^c^	Lower	Higher
Protocol duration	Shorter	Longer

a Omitting NaOH treatment, overall toxicity while performing comet-FISH is lowered.

b Rate of successful hybridization in five separate experiments on 30 nucleoids (mean % value ± standard deviation).

c Hotplate temperature is routinely checked but the transfer of heat onto slides cannot be determined.
